# Analysis of the related risk factors of inguinal lymph node metastasis in patients with penile cancer: A cross-sectional study

**DOI:** 10.1590/S1677-5538.IBJU.2021.0613

**Published:** 2022-01-10

**Authors:** Yatao Jia, Hongwei Zhao, Yun Hao, Jiang Zhu, Yingyi Li, Yanbo Wang

**Affiliations:** 1 Baoji People's Hospital Department of Urology Baoji Shaanxi China Department of Urology, Baoji People's Hospital, Baoji, Shaanxi, China; 2 The First Hospital of Jilin University Department of Urology Changchun Jilin China Department of Urology, The First Hospital of Jilin University, Changchun, Jilin, China; 3 The First Hospital of Jilin University Department of Neurology Changchun Jilin China Department of Neurology, The First Hospital of Jilin University, Changchun, Jilin, China; 4 Jilin University First Hospital Branch Department of Nephrology Changchun Jilin China Department of Nephrology, Jilin University First Hospital Branch, Changchun, Jilin, China

**Keywords:** Penile Neoplasms, Lymphatic Metastasis, Risk Factors

## Abstract

**Purpose:**

To determine independent predictors of inguinal lymph node (ILN) metastasis in patients with penile cancer.

**Patients and methods:**

We retrospectively analyzed all patients with penile cancer who underwent surgery at our medical center in the last ten years (n=157). Using univariate and multivariate logistic-regression models, we assessed associations with age, medical-history, phimosis, onset-time, number and maximum diameter of involved ILNs measured by imaging, pathological T stage, degree of tumor differentiation and/or cornification, lymphatic vascular infiltration (LVI), nerve infiltration, and ILN metastases. Interaction and stratified analyses were used to assess age, phimosis, onset time, number of ILNs, cornification, and nerve infiltration.

**Results:**

A total of 110 patients were included in the study. Multiple logistic regression analysis showed that the following factors were significantly correlated with ILN metastasis: maximum diameter of enlarged ILNs, T stage, pathological differentiation, and LVI. Among patients with a maximum ILN diameter ≥1.5cm, 50% had lymph node metastasis whereas 30.6% patients with a maximum ILN diameter <1.5cm showed LNM. Among 44 patients with stage Ta/T1, 10 showed ILN metastases, while 47.0% patients with stage T2 showed ILN metastases. Among 40 patients with highly differentiated penile-cancer, eight showed ILN metastasis, while 47.1% patients with low-to-middle differentiation showed ILN metastases. The rate of LNM was 33.3% in the LVI-free group and 64.3% in the LVI group.

**Conclusion:**

Our single-center results suggested that maximum ILN diameter, pathological T stage, pathological differentiation, and LVI were independent risk factors for ILN metastases.

## INTRODUCTION

Penile cancer is a rare malignancy, with an incidence of 0.081 per 100.000 in the United States and Europe (1, 2), and a prevalence of 2.3 to 8.3 per 100.000 in some developing regions, such as Asia, parts of Africa and Brazil (3, 4). Penile cancer is highly malignant and is mainly spread by lymphatic metastasis. The point of origin is the inguinal lymph nodes (ILNs), and jump metastasis rarely occurs (5, 6) ILN metastasis is the most important determinant of treatment and prognosis in patients with penile cancer (3, 7).

Pathology after lymph node biopsy or lymph node dissection remains the gold standard for the evaluation of ILN metastasis. However, it is an invasive operation that involves many postoperative complications, including poor lymph node drainage and poor wound healing (8). Therefore, researchers must explore the risk factors for ILN metastasis to determine which patients with penile cancer require ILN dissection. In so doing, patients with occult metastasis could receive prompt treatment, while patients with a lower risk of ILN metastasis could avoid excessive treatment.

Tumor stage, histological grade, lymphatic and vascular infiltration, histological subtype, and human papillomavirus have been identified as important predictors of ILN metastasis in previous studies (9, 10). However, these studies were conducted in a single center, and the sample size was small. In addition, only univariate analysis was used to explore the risk factors for ILN metastasis. In the present study, we aimed to test the independent risk factors and the role of inguinal lymph node metastasis in penile cancer in different populations, especially in China. We conducted multiple logistic and subgroup analyses, and interaction tests on all patients with penile cancer who underwent surgery in a large tertiary hospital over a 10-year period.

## MATERIALS AND METHODS

This cross-sectional study was conducted between January 2010 and December 2019 at a comprehensive tertiary hospital in China. The inclusion criteria were as follows: (1) primary tumor treated surgically, (2) tumor pathology confirmed by experienced pathologists, and (3) ILN metastasis pathologically confirmed by biopsy or prophylactic inguinal lymphadenectomy. Patients with pelvic lymph node or distant metastases were excluded, as were those treated in other hospitals. All patients provided informed consent and the institutional ethics committee approved the study (IRB-032-06).

We retrieved the following clinical information from the patient's medical records: age, previous medical history (hypertension, diabetes, or cardiovascular disease), phimosis, onset time, number and maximum diameter of the involved ILNs, pathological T stage, degree of tumor differentiation and/or cornification, lymphatic vascular infiltration (LVI), and nerve infiltration. The number and maximum diameter of the ILNs were determined using ultrasound or computed tomography. Tumor stage was assessed according to the TNM Classification of Malignant Tumors (TNM) system, which was updated in 2018 (11). The pathological differentiation of tumors was evaluated according to the criteria described by Velazquez et al. (12).

Primary tumors were treated surgically using either penis-sparing, partial amputation, or total excision via perineal urethrostomy. According to the EAU guidelines (2009) (13), ILN biopsy or bilateral ILN dissection is recommended for patients with stage ≥T1G2 and/or enlarged lymph nodes that have not significantly shrunk after 4-6 weeks of antibiotic treatment. Surgery was performed by three experienced urologists.

Continuous variables are presented as mean±standard deviation (SD) or median (interquartile range), while categorical variables are expressed as frequencies or percentages. To facilitate statistical analysis, all continuous variables except age were converted into categorical variables. Risk factors for penile cancer were identified using univariate logistic regression analyses, and independent predictive factors for ILN metastasis were confirmed by multivariate logistic regression analyses. The statistical packages R (The R Foundation, Vienna, Austria) was used to analyze the data. Statistical differences were considered significant when the P-value was less than 0.05.

## RESULTS

**Figure 1:**
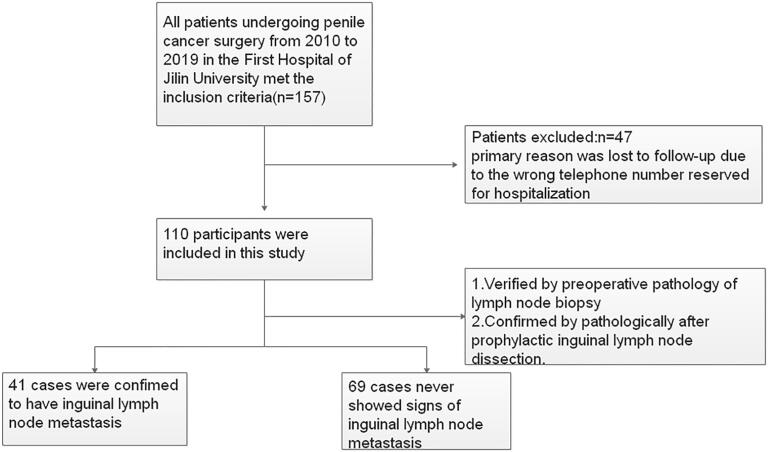
Flowchart for the recruitment of patients in this study

A total of 157 patients were identified; 47 were excluded due to lack of follow-up data, resulting in 110 patients included in the study (Figure-1). Forty-one patients had confirmed ILN metastasis, of whom 25 were pathologically confirmed by ILN biopsy and the rest were confirmed by pathology after prophylactic inguinal lymphadenectomy. There were no signs of ILN metastasis in 69 patients. Hence, the rate of ILN metastasis was 37.3%.

Table-1 lists the clinicopathological characteristics and univariate analysis of the variables associated with ILN metastasis in the 110 patients. The mean age was 61.6±11.8 years. Univariate analysis showed that the following factors were correlated with ILN metastasis: maximum diameter of enlarged ILNs (P=0.045), pathological stage (P=0.010), degree of pathological differentiation (P=0.009), and LVI (P=0.025).

**Table 1:** Clinicopathological characteristics and univariate analysis of variables associated with inguinal lymph node metastasis

	Total	Without metastasis	With metastasis	HR(95%CI)	P-value
N	110	69	41		
Age(years)(Mean+SD)	61.6 ± 11.8	61.0 ± 12.0	62.5 ± 11.5	0.1 (-0.3, 0.5)	0.529
HBP(N,%)				0.0 (-0.4, 0.4)	0.984
no	94 (85.5%)	59 (85.5%)	35 (85.4%)		
yes	16 (14.5%)	10 (14.5%)	6 (14.6%)		
Diabetes(N,%)				0.1 (-0.3, 0.4)	0.799
no	101 (91.8%)	63 (91.3%)	38 (92.7%)		
yes	9 (8.2%)	6 (8.7%)	3 (7.3%)		
CDV(N,%)				0.1 (-0.2, 0.5)	0.439
no	102 (92.7%)	65 (94.2%)	37 (90.2%)		
yes	8 (7.3%)	4 (5.8%)	4 (9.8%)		
Phimosis(N,%)				0.2 (-0.2, 0.6)	0.347
no	58 (52.7%)	34 (49.3%)	24 (58.5%)		
yes	52 (47.3%)	35 (50.7%)	17 (41.5%)		
Onset_time(month) (N,%)				0.1 (-0.2, 0.5)	0.475
<12	76 (69.1%)	46 (66.7%)	30 (73.2%)		
>=12	34 (30.9%)	23 (33.3%)	11 (26.8%)		
Number_ILN(N,%)				0.2 (-0.2, 0.6)	0.254
≥3	46 (41.8%)	26 (37.7%)	20 (48.8%)		
>3	64 (58.2%)	43 (62.3%)	21 (51.2%)		
Maxium_ILN(N,%)				0.4 (0.0, 0.8)	0.045
>1.5cm	72 (65.5%)	50 (72.5%)	22 (53.7%)		
≥1.5cm	38 (34.5%)	19 (27.5%)	19 (46.3%)		
T_stage(N,%)				0.5 (0.1, 0.9)	0.01
Ta/T1	44 (40.0%)	34 (49.3%)	10 (24.4%)		
T2 and higher	66 (60.0%)	35 (50.7%)	31 (75.6%)		
Differentiation(N,%)				0.6 (0.2, 1.0)	0.005
lower-middle	70 (63.6%)	37 (53.6%)	33 (80.5%)		
higher	40 (36.4%)	32 (46.4%)	8 (19.5%)		
Cornification(N,%)				0.0 (-0.3, 0.4)	0.807
no	74 (67.3%)	47 (68.1%)	27 (65.9%)		
yes	36 (32.7%)	22 (31.9%)	14 (34.1%)		
LVI(N,%)				0.4 (0.0, 0.8)	0.025
no	96 (87.3%)	64 (92.8%)	32 (78.0%)		
yes	14 (12.7%)	5 (7.2%)	9 (22.0%)		
Nerve_infiltration(N,%)				0.2 (-0.2, 0.6)	0.362
no	92 (83.6%)	56 (81.2%)	36 (87.8%)		
yes	18 (16.4%)	13 (18.8%)	5 (12.2%)		

The P value is written in italics when it is less than 0.05

HBP, high blood pressure; CDV, cardiovascular disease; ILN, inguinal lymph node; LVI, lymphatic vascular infiltration, T stage, the TNM system of penile cancer updated in 2018; degree of tumor differentiation: According to the percentage of undifferentiated cells, the tumor was divided into middle and low differentiated groups and highly differentiated groups. CI, confidence interval; HR, hazard ratio.

Significant single factors were included in the multivariate analysis (Table-2). We applied both non-adjusted and multivariate adjusted models (adjusted for age, previous medical history, and other variables that affected the X regression coefficient by more than 10%). A two-sided significance level of 0.05 was used to evaluate statistical significance. The results showed that the following factors were independent predictors of ILN metastasis: largest diameter of enlarged ILNs, T stage of tumor, pathological differentiation, and LVI. Specifically, patients with the largest ILN diameter ≥1.5cm showed a 1.3-fold increased risk of metastasis compared to those with the largest ILN diameter <1.5cm. Those with tumor stage T2 and above showed a two-fold greater risk of ILN metastasis than those with tumor stage Ta or T1. Those with low to moderate tumor differentiation had a 2.6-fold greater risk of ILN metastasis than those with high pathological differentiation. Finally, patients with LVI had a 2.6-fold greater risk of ILN metastasis than those without LVI.

**Table 2:** Multiple logistic regression models assessed the correlation between risk factors and ILN metastasis

Exposure		Non-adjusted	Adjust I	Adjust II
		HR,95%CI P value	HR,95%CI P value	HR,95%CI P value
Maxium_ILN				
	<1.5cm	1	1	1
	≥1.5cm	2.3 (1.0, 5.1) 0.047	2.4 (1.1, 5.6) 0.035	10.7 (2.1, 53.3) 0.004
T_stage				
	Ta/T1	1	1	1
	T2 and above	3.0 (1.3, 7.1) 0.011	3.1 (1.3, 7.5) 0.010	7.1 (1.7, 28.9) 0.006
Differentiation				
	high	1	1	1
	low-middle	3.6 (1.4, 8.8) 0.006	4.0 (1.5, 10.4) 0.004	6.2 (1.9, 20.2) 0.003
LVI				
	no	1	1	1
	yes	3.6 (1.1, 11.6) 0.032	3.6 (1.1, 11.8) 0.033	7.4 (1.3, 40.8) 0.022

The data in the table: β (95%CI) P value/OR (95%CI) P value Outcome variable: metastasis Exposed variables: **1) Maximum_ILN** Non-adjusted model adjusted for: None Adjust I model adjust for: age; HBP; diabetes; CDV Adjust II model adjusted for: model1+onset_time; T_stage; differentiation; LVI; umber_ILN **2) T_stage** Non-adjusted model adjusted for: None Adjust I model adjusted for: age; HBP; diabetes; CDV Adjust II model adjusted for: model1+onset_time; Number_ILN; Maximum_ILN; differentiation; LVI; nerve infiltration **3) Differentiation** Non-adjusted model adjusted for: None Adjust I model adjusted for: age; HBP; diabetes; CDV Adjust II model adjusted for: model1+onset_time; Number_ILN; Nerve_infiltration **4) LVI** Non-adjusted model adjusted for: None Adjust I model adjusted for: age; HBP; diabetes; CDV Adjust II model adjusted for: model1+onset_time, Number_ILN, Maximum_ILN, T_stage, Nerve_infiltration, differentiation ILN, inguinal lymph nodes; LVI, lymphatic vascular infiltration.

**Figure 2:**
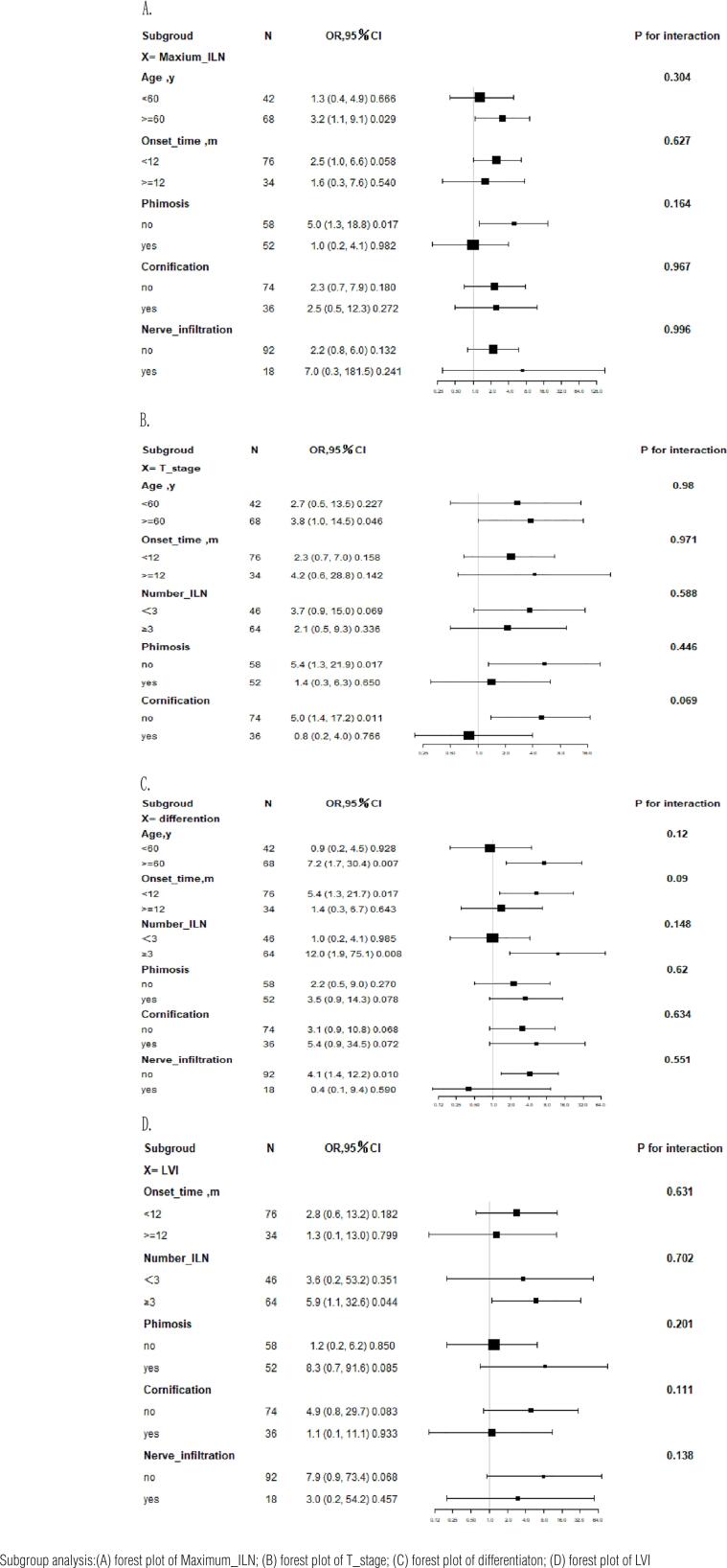
Odds ratios for inguinal lymph node metastasis, according to baseline characteristics

To further demonstrate the stability of our results, we performed stratified analyses and interaction tests of the four independent risk factors, as shown in the forest plot (Figure-2a-d and Table-3). The results showed that no significant interactions were observed.

**Table 3:** Association between related risk factors and inguinal lymph node metastasis, according to baseline characteristics.

Subgroud		N	OR,95%CI	P value	P for interaction
X= Maxium_ILN					
Age, y					0.304
	<60	42	1.3 (0.4, 4.9)	0.666	
	>=60	68	3.2 (1.1, 9.1)	0.029	
Onset_time, m					0.627
	<12	76	2.5 (1.0, 6.6)	0.058	
	>=12	34	1.6 (0.3, 7.6)	0.54	
Phimosis					0.164
	no	58	5.0 (1.3, 18.8)	0.017	
	yes	52	1.0 (0.2, 4.1)	0.982	
Cornification					0.967
	no	74	2.3 (0.7, 7.9)	0.18	
	yes	36	2.5 (0.5, 12.3)	0.272	
Nerve_infiltration					0.996
	no	92	2.2 (0.8, 6.0)	0.132	
	yes	18	7.0 (0.3, 181.5)	0.241	
X= T_stage					
	Age, y				0.98
	<60	42	2.7 (0.5, 13.5)	0.227	
	>=60	68	3.8 (1.0, 14.5)	0.046	
Onset_time, m					0.971
	<12	76	2.3 (0.7, 7.0)	0.158	
	<=12	34	4.2 (0.6, 28.8)	0.142	
Number_ILN					0.588
	<3	46	3.7 (0.9, 15.0)	0.069	
	≥3	64	2.1 (0.5, 9.3)	0.336	
Phimosis					0.446
	no	58	5.4 (1.3, 21.9)	0.017	
	yes	52	1.4 (0.3, 6.3)	0.65	
Cornification					0.069
	no	74	5.0 (1.4, 17.2)	0.011	
	yes	36	0.8 (0.2, 4.0)	0.766	
X= differention					
Age,y					0.12
	<60	42	0.9 (0.2, 4.5)	0.928	
	>=60	68	7.2 (1.7, 30.4)	0.007	
Onset_time,m					0.09
	<12	76	5.4 (1.3, 21.7)	0.017	
	>=12	34	1.4 (0.3, 6.7)	0.643	
Number_ILN					0.148
	<3	46	1.0 (0.2, 4.1)	0.985	
	≥3	64	12.0 (1.9, 75.1)	0.008	
Phimosis					0.62
	no	58	2.2 (0.5, 9.0)	0.27	
	yes	52	3.5 (0.9, 14.3)	0.078	
Cornification					0.634
	no	74	3.1 (0.9, 10.8)	0.068	
	yes	36	5.4 (0.9, 34.5)	0.072	
Nerve_infiltration					0.551
	no	92	4.1 (1.4, 12.2)	0.01	
	yes	18	0.4 (0.1, 9.4)	0.59	
X= LVI					
Onset_time, m					0.631
	<12	76	2.8 (0.6, 13.2)	0.182	
	>=12	34	1.3 (0.1, 13.0)	0.799	
Number_ILN					0.702
	<3	46	3.6 (0.2, 53.2)	0.351	
	≥3	64	5.9 (1.1, 32.6)	0.044	
Phimosis					0.201
	no	58	1.2 (0.2, 6.2)	0.85	
	yes	52	8.3 (0.7, 91.6)	0.085	
Cornification					0.111
	no	74	4.9 (0.8, 29.7)	0.083	
	yes	36	1.1 (0.1, 11.1)	0.933	
Nerve_infiltration					0.138
	no	92	7.9 (0.9, 73.4)	0.068	
	yes	18	3.0 (0.2, 54.2)	0.457	

OR, odds ratio; CI, confidence interval.

## DISCUSSION

The most important factor affecting the prognosis of penile cancer is ILN metastasis (3, 14–16). Several studies have indicated that the rate of ILN metastasis in patients with penile cancer is 30–40% (17). The latest meta-analysis (18) selected 42 eligible studies that included a total of 4.802 patients, of whom 1.706 (36%) were diagnosed with ILN metastasis. This finding was corroborated by our results, in which 37.3% of the patients had ILN metastasis (41/110).

In our multiple regression analysis, the maximum diameter of the enlarged ILNs, pathological stage, pathological differentiation, and LVI were the only predictors of ILN metastasis. We conducted a stratified analysis and interaction tests on the four factors and found no any obvious interaction, further proving the stability of our results.

A maximum ILN diameter of >1.0cm is usually considered abnormal, while a diameter >1.5cm, with a relatively hard texture, strongly indicates tumor metastasis (19). The present study suggested that the largest diameter of enlarged ILNs was an independent risk factor, corroborating studies by Tang et al. (20) and Zhou et al. (9). However, in another study, 50% of enlarged ILNs were inflamed or reactive, rather than metastatic (17), indicating that ILN metastasis cannot be reliably detected using imaging or clinical evaluation. It is essential to predict ILNM in combination with the pathological characteristics of the primary tumor.

The pathological stage of the primary tumor is generally considered the most important parameter for predicting ILN metastasis in patients with penile cancer (21). Depending on whether there is infiltration of the urethra or corpus cavernosum, the tumor stage can be classified as T1, T2, or higher (11). In the present study, the rate of ILN metastasis in patients with T1 stage was 22.7% (10/44), while it was 47.0% (31/66) in those with T2 stage and above. The ILN metastasis rate in patients with T2 stage disease was significantly higher than that in patients with T1 stage disease. Several researchers have suggested that if the corpus cavernosum is infiltrated, ILN dissection should be performed, even if there are no obvious enlarged ILNs (22, 23). However, deciding to perform ILN dissection in all patients with T2 and above based only in tumor stage will lead to overtreatment (24). In the present study, 53.0% of patients with T2 stage disease and above showed no obvious signs of ILN metastasis. Therefore, other factors should be considered when screening for high-risk patients.

The degree of tumor differentiation under pathological conditions is negatively correlated with tumor pathological grade or malignancy, with lower tumor differentiation indicating higher grade, higher malignancy, and greater risk of metastasis (18). Solsona et al. (25) found that the grade of tumor differentiation correlates well with ILN metastasis, corroborating our multiple regression analysis (HR=3.0, 95% CI: 1.3-7.1). According to Horenblas et al. (26), the lymph node metastasis rates of G1, G2, and G3 were 29%, 46%, and 82%, respectively. Our results were similar to these, with 20% (8/40), 45% (30/66), and 75% (3/4), respectively. Theodoresu et al. (27) asserted that tumor grade is the only predictor of ILN metastasis, and they recommended ILN dissection in patients with G2 and G3, which coincides with our point of view.

The existing literature has different opinions on whether LVI is a predictor of ILN metastasis. Some studies have ranked LVI as one of the most important factors of metastasis (28–31), while others have not (32). Our findings suggest that LVI is significantly associated with ILN metastasis (HR=3.6, 95% CI: 1.1-11.6) among patients with penile cancer.

The present study had several strengths. First, although some potential confounding factors were unavoidable, we used strict statistical adjustments to minimize residual confounding. Second, the effect modifier factor analysis took full advantage of the data; no interaction was found, indicating that the results were more stable.

However, there were some limitations to our study. Patients with penile cancer were recruited from a large single medical center. Therefore, an external validation of the results is required. The study was cross-sectional, and no information was available about the degree of risk factors prior to ILN metastasis, because the earlier pathology reports contained no such data. Moreover, no data were available regarding lymph node extranodal transfer and the growth pattern of tumors in some patients (papillary, ulcerated, invasive,) therefore, we could not consider these variables in the final results, although there was evidence that these factors had prognostic significance. Despite these limitations, the findings of this study have implications for clinicians when formulating further treatment plans for patients with penile cancer who have undergone surgery. However, prospective studies with a larger sample size are required.

## CONCLUSION

In conclusion, the maximum diameter of the enlarged ILN, pathological stage, pathological differentiation, and LVI were independent predictive factors that worsened the prognosis of patients with penile cancer. Specifically, patients with enlarged lymph nodes >1.5cm in diameter, pathological stage T2 and above, low-to-middle differentiation, and LVI are more likely to develop ILN metastasis. Prophylactic ILN dissection is recommended for these patients. Prospective studies with larger sample sizes are required to support our findings.

## ABBREVIATIONS

ILN: inguinal lymph node;
LVI: lymphatic vascular infiltration;
SD: standard deviation;
HBP: high blood pressure;
CDV: cardiovascular disease;
CI: confidence interval;
HR: hazard ratio.
